# Computed Tomography Scan Overestimates the Size of Pericardial Effusion Compared to Echocardiography

**DOI:** 10.7759/cureus.5182

**Published:** 2019-07-20

**Authors:** Abdelmoniem Moustafa, Xinlu Liu, Feng Ye, Sadik Khuder, Luai Alhazmi, Eslam Youssef, Hussam Alim, Mohd Amer Alsamman, Mohammad S Khan, Ehab Eltahawy

**Affiliations:** 1 Internal Medicine, The Miriam Hospital, Brown University, Providence, USA; 2 Internal Medicine, Prisma Health, Columbia, USA; 3 Internal Medicine, University of Toledo Medical Center, Toledo, USA; 4 Public Health, University of Toledo Medical Center, Toledo, USA; 5 Cardiology, University of Toledo Medical Center, Toledo, USA; 6 Radiology, University of Toledo Medical Center, Toledo, USA; 7 Internal Medicine, The Miriam Hospital, Providence, USA

**Keywords:** computed tomography, echocardiography, accuracy, pericardial effusion, computed tomography

## Abstract

Objective: Pericardial effusion is not an uncommon finding in hospitalized patients. Many pericardial effusions are found incidentally through computed tomography (CT) performed for other indications. Echocardiography is usually ordered when an incidentally discovered pericardial effusion is found on the CT to examine the effect of the effusion on hemodynamics and to detect early signs of tamponade. However, in clinical practice, the discrepancy between CT and echocardiography regarding the size of pericardial effusions is common. The accuracy of CT in the evaluation of the size of pericardial effusions is not well-studied. Our study aims to evaluate the accuracy of CT in assessing the size of a pericardial effusion compared with the gold standard echocardiography.

Methods: This is a retrospective study examining patients presenting to the University of Toledo Medical Center (UTMC) with pericardial effusions. One hundred and forty-one patient charts were reviewed and 45 subjects were excluded. Ninety-six patients in whom both CT and echocardiography were performed were enrolled in the final analysis. The time interval between both imaging modalities was limited to less than 14 days and no interventions on the effusion (e.g., pericardiocentesis) occurred in the time interval between the two imaging modalities.

Results: The size of the pericardial effusion was assessed similarly between CT and echocardiography in 50% of the cases (48/96). In the other half of the study population, the results were discrepant; CT was found to overestimate the size of pericardial effusion in 44% of the cases (42/96). The agreement rate between the two modalities is significantly low kappa = 0.111, P = 0.028.

The independent variables age, gender, body mass index (BMI), use of anticoagulants, and renal function had no effect on the agreement between CT and echocardiography.

Conclusion: Computerized tomography tends to overestimate the size of the pericardial effusion compared to echocardiography. Based on an incidental finding of pericardial effusion on CT scan, this discrepancy should be recognized prior to ordering an echocardiogram. Echocardiography can be considered in relevant clinical settings.

## Introduction

The pericardium is the fibroelastic sac surrounding the cardiac muscle and the great vessels. The main function of the pericardium is preventing overdistention of the ventricles [1-2]. Given that the pericardium has limited compliance, even small amounts of additional fluid can cause a sharp rise in pericardial pressure that may result in hemodynamic compromise. However, the slow accumulation of pericardial fluid can stretch the pericardium without circulatory collapse [2].

Pericardial effusions can be classified according to their size into small (< 10 mm), moderate (10 - 20 mm), and large (> 20 mm) on echocardiographic imaging [1-3]. In terms of pericardial effusion volume, a small effusion on echocardiography (< 10 mm) correlates with 50 - 250 ml of fluid, moderate (10 - 20 mm) correlates with 250 - 500 ml, and > 500 ml for a large effusion [4].

Although small and moderate chronic effusions can be managed expectantly with serial echocardiography, large pericardial effusions are associated with an elevated risk for hemodynamic compromise and may require intervention; one-third of these patients progress to cardiac tamponade. Therefore, in large pericardial effusions, particularly with symptoms or signs of early hemodynamic compromise, pericardiocentesis or surgical drainage is recommended [1-2, 5-6].

Many pericardial effusions are incidentally found on computed tomography (CT) when performed for a different purpose. An echocardiogram is often subsequently ordered, sometimes urgently, due to its superiority in the characterization of how these effusions affect hemodynamics. In other instances, CT has been used following echocardiography to further assess pericardial effusions regarding traits, such as transudate versus exudative, clots, pericardial fat, and loculations [5, 7-9].

One concern that arises in the use of both modalities is the discrepancy in the assessment of the size of the pericardial effusion (small, moderate, and large). This was evidenced in a previous study involving follow-up of post-transcatheter aortic valve implantation (TAVI) patients in whom pericardial effusion was one of the post-procedural complications monitored [10]. In this study, 38/143 (26%) patients who received echocardiography and CT had discordant assessments of the size of the effusion on the two separate modalities.

According to the American Society of Echocardiography, echocardiography is the gold standard imaging modality for detection of pericardial effusion and evaluation of the physiologic and hemodynamic effects of the effusion [7]. However, the prevalence of pericardial effusion found incidentally on CT scans is as high as 5%. This finding often results in concern on the part of the patient, as well as the ordering physician, and may lead to a cascade of further testing, including echocardiograms. With this in mind, we sought to examine the concordance of CT scan and echocardiography in assessing the size of pericardial effusions.

## Materials and methods

Patient selection

Data were extracted from the database of the Radiology Department at the University of Toledo Medical Center (UTMC). All inpatients who had a pericardial effusion on CT from January 2012 and September 2017 and had an echocardiogram either before or after the CT scan were included. There could not have been more than a 14-day interval between the two imaging modalities for the patient to be included.

**Figure 1:**
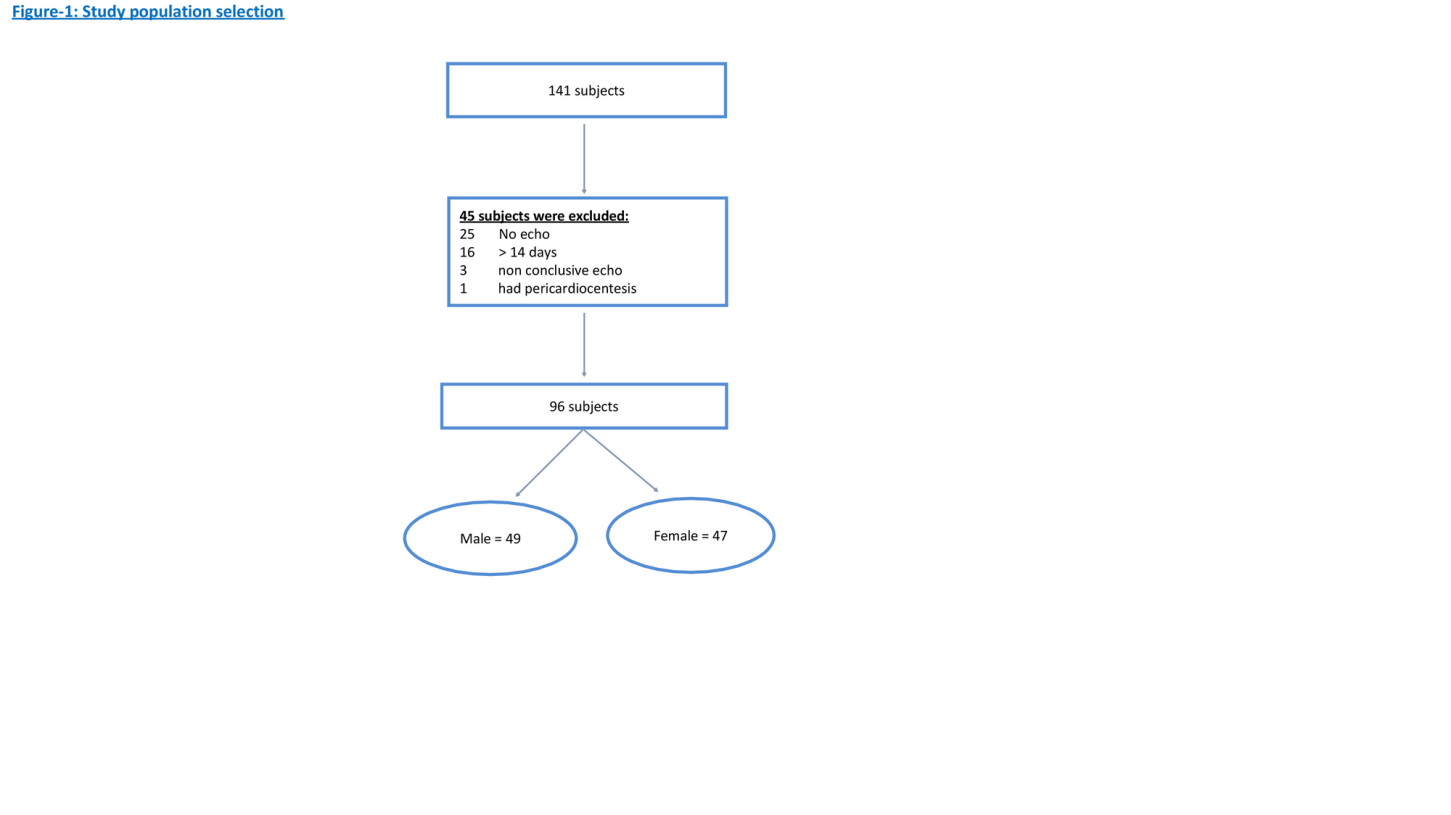
Breakdown analysis of 141 potential subjects to 96 patients accepted in the study

The size of the pericardial effusion reported on CT evaluation was compared to the size of the pericardial effusion on the echocardiogram report. Of the 141 potential patients identified, 45 were excluded (25 patients had no echocardiogram, in 16 patients there was more than a 14-day interval between both imaging modalities, three patients were excluded as the echocardiogram report was inconclusive (pericardial effusion vs. fat pad), and one patient was excluded as pericardiocentesis was performed prior to the second imaging modality). This resulted in data from 96 patients that were analyzed (Figure 1). Patients’ demographics, co-morbidities, medications at admission, baseline laboratory values, and echocardiographic findings were extracted from the chart review. The Institutional Review Board at UTMC approved the study with a waiver of informed consent.

Computed tomography

The size of the pericardial effusion was assessed from the CT reports generated by the Radiology Department at UTMC. The size of the pericardial effusion fell in one of four categories: small to moderate, moderate, moderate to large, and large.

Echocardiography

The size of effusion on the echocardiogram was considered small if < 1 cm, moderate if between 1 - 2 cm, and large if more than 2 cm. The size of the effusion was obtained from the echocardiographic reports generated by the Division of Cardiovascular Medicine. If the report did not provide a definitive assessment for the size of the effusion, the study was reexamined and an assessment of the size obtained at the parasternal long-axis view. The reader of the echocardiogram in such cases was blinded to the results of the CT. In addition to the size of the effusion, data on the ejection fraction was also collected.

Data analysis

When the size of the effusion on CT was reported as ‘small to moderate’, it was considered matching with the echocardiography if the size on echocardiography was either mild or moderate. The same rule was applied for the moderate to a large category on the CT. 

Endpoints

The primary endpoint is the percent agreement between the size of the pericardial effusion on the CT and echocardiography. The secondary end-point was the effect of patients’ characteristics and other echocardiographic findings on the agreement between the two imaging modalities.

Statistical analysis

The agreement between the size of the pericardial effusion reported on the CT compared to echocardiography was examined using kappa statistics. A binary logistic regression was used to test the effect of age, gender, body mass index (BMI), and serum creatinine on the correlation between the two imaging modalities.

## Results

Patient characteristics

A total of 96 patients (mean age: 60, standard deviation (SD) +/- 16) were identified and analyzed. A total of 51% of male patients and 74% of female patients had a BMI > 25. Serum creatinine > 1.2 was found in 43% of the overall study population (Table 1).

**Table 1:** Study Population Characteristics BMI: body mass index; SD: standard deviation

	Mean	SD	Range
Age	60	16	19 - 92
BMI	30	9.3	16 - 75
Serum creatinine	2.16	2.6	0.3 - 15.5

Echocardiogram

The majority (46%) of the pericardial effusions were small effusions, and out of 96 patients, only 19 (19%) had a large pericardial effusion. Sixteen of the 96 patients (16.6%) had left ventricular (LV) dysfunction (ejection fraction < 45%). Fifty percent of the patients had the echocardiogram done prior to CT (Table 2).

**Table 2:** Different Sizes of Pericardial Effusion on Echocardiography

	Frequency	Percent	Cumulative frequency	Cumulative percent
None	7	7.29	7	7.29
Small	44	45.83	51	53.13
Moderate	26	27.08	77	80.21
Large	19	19.79	96	100

Computed tomography

Moderate effusion was the most frequently reported finding on the CT scan. On the other hand, large pericardial effusions represented only 28% (n = 27) of the findings (Table 3).

**Table 3:** Different Sizes of Pericardial Effusion on Computed Tomography

	Frequency	Percent	Cumulative frequency	Cumulative percent
Small to moderate	23	23.96	23	23.96
Moderate	44	45.83	67	69.79
Moderate to large	6	6.25	73	76.04
Large	23	23.96	96	100

Agreement between CT and echocardiography

The size of pericardial effusion correlated well between the two imaging modalities in 50% of the cases (48/96). In the other half of the study population where the results did not match, the CT was found to overestimate the size of the pericardial effusion in 44% of the cases (42/96). Small effusions had the highest agreement rate between the two modalities. On the other hand, moderate effusions had the lowest agreement rate. The agreement rate between the two modalities was significantly low kappa = 0.111, P = 0.028 (Table 4).

**Table 4:** Agreement Between Two Imaging Modalities CT: computed tomography; echo: echocardiography

		None (Echo)	Mild (Echo)	Moderate (Echo)	Large (Echo)	Total
Small to moderate (CT)	Frequency	1	18	3	1	23
	Percent	1.06%	18.75%	3.13%	1.04%	23.96%
	Row percent	4.35%	78.26%	13.04%	4.35%	
	Column percent	14.29%	40.91%	11.54%	5.26%	
Moderate (CT)	Frequency	6	22	11	5	44
	Percent	6.25%	22.92%	11.46%	5.21%	45.83%
	Row percent	13.64%	50%	25%	11.36%	
	Column percent	85.71%	50%	42.31%	26.32%	
Moderate to large (CT)	Frequency	0	2	2	2	6
	Percent		2.08%	2.08%	2.08%	6.25%
	Row percent		33.33%	33.33%	33.33%	
	Column percent		4.55%	7.69%	10.53%	
Large (CT)	Frequency	0	2	10	11	23
	Percent		2.08%	10.42%	11.46%	23.96
	Row percent		8.70%	43.48%	47.83%	
	Column percent		4.55%	38.46%	57.89%	
Total		7 (7.29%)	44 (45.83%)	26 (27.08%)	19 (19.79%)	96 (100%)

Age, gender, BMI, and use of anticoagulant renal function had no effect on the agreement between the CT and echocardiogram.

## Discussion

In this study, the relationship between CT and echocardiographic evaluation of the size of pericardial effusions was examined. When compared to the assessment of size by echocardiography, 1) CT assessed 50% of pericardial effusions similarly, 2) CT overestimated the size of pericardial effusions in 44% of patients, and 3) clinical variables, including BMI, kidney function, status of anticoagulation, left ventricular systolic function, and right ventricular systolic pressure, did not affect the accuracy of the CT.

Despite these discrepancies, a previous study showed that CT was a valuable complementary modality to echocardiography in the evaluation of pericardial effusion [11]. CT is advantageous in imaging focal effusions, attenuated values of pericardial fluid (Hounsfield units) on CT provides additional information about the nature of the fluid, and CT identifies pericardial thickness, inflammation, and calcification. Current multidetector CT scanners can provide high-resolution anatomical detail of the heart, pericardium, and adjacent structures, which can be used to diagnose congenital pericardial cysts and neoplastic disease with direct pericardial invasion or metastasis to pericardium [12].

The prognosis of pericardial effusion is associated with the etiology and the size. In general, bacterial and neoplastic etiologies have a poorer prognosis. Small to moderate idiopathic pericardial effusions have relatively benign outcomes in most cases, whereas large pericardial effusions are associated with the potential for hemodynamic compromise and cardiac tamponade. In our study, CT overestimates the size of pericardial effusions in 44% of patients, and in the 6% of cases, CT underestimated the pericardial effusion size.

Computerized tomography tends to overestimate the size of the pericardial effusion compared to echocardiography. Based on an incidental finding of pericardial effusion on CT scan, this discrepancy should be recognized prior to ordering an echocardiogram. Echocardiography can be considered in relevant clinical settings.

Study limitations

Several limitations of the study exist. This was a retrospective study from a single tertiary referral center with potential selection and referral bias. The sample size was relatively small and without the long-term follow-up of patient outcomes. Almost 50% of the subjects had echocardiography performed prior to CT; this may have influenced the radiologists’ assessment of the size of the pericardial effusion as they were not blinded to the results of the prior echocardiogram. Finally, the duration of time between CT and echocardiography may be of sufficient length to have affected the size of the effusion between the studies and hence affecting the accuracy of the comparison.

## Conclusions

In conclusion, CT assessment of the size of pericardial effusions in asymptomatic patients lacks accuracy. In patients with CT-diagnosed small-to-moderate pericardial effusions, echocardiography is generally not required urgently. Echocardiography may be very useful in the subsequent workup of presence, size, and etiology of the effusion. In patients in whom CT demonstrates a large pericardial effusion, it is an overestimation 50% of the time. Therefore, the patient’s clinical condition should determine the timing of further echocardiographic evaluation rather than the size of effusion on CT.
